# Spatial Variation of Leaf Optical Properties in a Boreal Forest Is Influenced by Species and Light Environment

**DOI:** 10.3389/fpls.2017.00309

**Published:** 2017-03-14

**Authors:** Jon Atherton, Beñat Olascoaga, Luis Alonso, Albert Porcar-Castell

**Affiliations:** ^1^Optics of Photosynthesis Laboratory, Department of Forest Sciences, Viikki Plant Science Center, University of HelsinkiHelsinki, Finland; ^2^Image Processing Laboratory, Department of Physics, University of ValenciaValencia, Spain

**Keywords:** chlorophyll fluorescence, leaf optical properties, photosynthesis, PRI, baseline

## Abstract

Leaf Optical Properties (LOPs) convey information relating to temporally dynamic photosynthetic activity and biochemistry. LOPs are also sensitive to variability in anatomically related traits such as Specific Leaf Area (SLA), via the interplay of intra-leaf light scattering and absorption processes. Therefore, variability in such traits, which may demonstrate little plasticity over time, potentially disrupts remote sensing estimates of photosynthesis or biochemistry across space. To help to disentangle the various factors that contribute to the variability of LOPs, we defined baseline variation as variation in LOPs that occurs across space, but not time. Next we hypothesized that there were two main controls of potentially disruptive baseline spatial variability of photosynthetically-related LOPs at our boreal forest site: light environment and species. We measured photosynthetically-related LOPs in conjunction with morphological, biochemical, and photosynthetic leaf traits during summer and across selected boreal tree species and vertical gradients in light environment. We then conducted a detailed correlation analysis to disentangle the spatial factors that control baseline variability of leaf traits and, resultantly, LOPs. Baseline spatial variability of the Photochemical Reflectance Index (PRI) was strongly influenced by species and to a lesser extent light environment. Baseline variability of spectral fluorescence derived LOPs was less influenced by species; however at longer near-infrared wavelengths, light environment was an important control. In summary, remote sensing of chlorophyll fluorescence has good potential to detect variation in photosynthetic performance across space in boreal forests given reduced sensitivity to species related baseline variability in comparison to the PRI. Our results also imply that spatially coarse remote sensing observations are potentially unrepresentative of the full scope of natural variation that occurs within a boreal forest.

## Introduction

Global and regional scale estimates of terrestrial primary productivity are made possible by optical remote sensing measurements of plant canopies. The state of the art in such measurements is moving toward the replacement of traditional broadband indices, such as the Normalized Difference Vegetation Index and Enhanced Vegetation Index, with potentially more responsive measures of short-term physiological activity. These physiologically related signals include the emission of solar-induced chlorophyll fluorescence (SIF) (Joiner et al., 2013; Frankenberg et al., 2014; Guanter et al., 2014; Zhang et al., 2016), and the Photochemical Reflectance Index (PRI), a narrow-band reflectance index that is sensitive to both rapid, sub-daily timescale and longer, seasonal timescale changes in carotenoid pigment contents (Gamon et al., 1992; Filella et al., 2009; Gamon and Berry, 2012; Porcar-Castell et al., 2012; Wong and Gamon, 2015).

An understanding of leaf scale processes is critical to the correct interpretation of remote sensing measurements of plants. This is because it is the Leaf Optical Properties (LOPs) that typically generates the signal of interest (e.g. SIF, PRI) in remote sensing data. By evaluating the links between LOPs and leaf photosynthetic and biochemical traits, we remove the dependence of LOPs on confounding canopy structural effects which potentially influence remote sensing estimations of traits at larger scales (Knyazikhin et al., 2013).

As with photosynthetic and biochemical leaf traits such as gas exchange parameters or pigment concentrations, photosynthetically-related LOPs vary across species and light gradients, and also in response to the spatial and temporal dynamics of environmental forcing factors such as temperature fluctuations or water availability. The covariation of LOPs with photosynthetic and biochemical traits underpins the basis of empirical remote sensing in which regression models are used to estimate trait values from optical measurements (Sims and Gamon, 2002; Serbin et al., 2014). However, LOPs are also influenced by leaf traits that do not necessarily co-vary with short-term changes in photosynthetic functionality or biochemistry, but do contribute to baseline spatial variability, which could potentially confound models.

**Figure 1:**
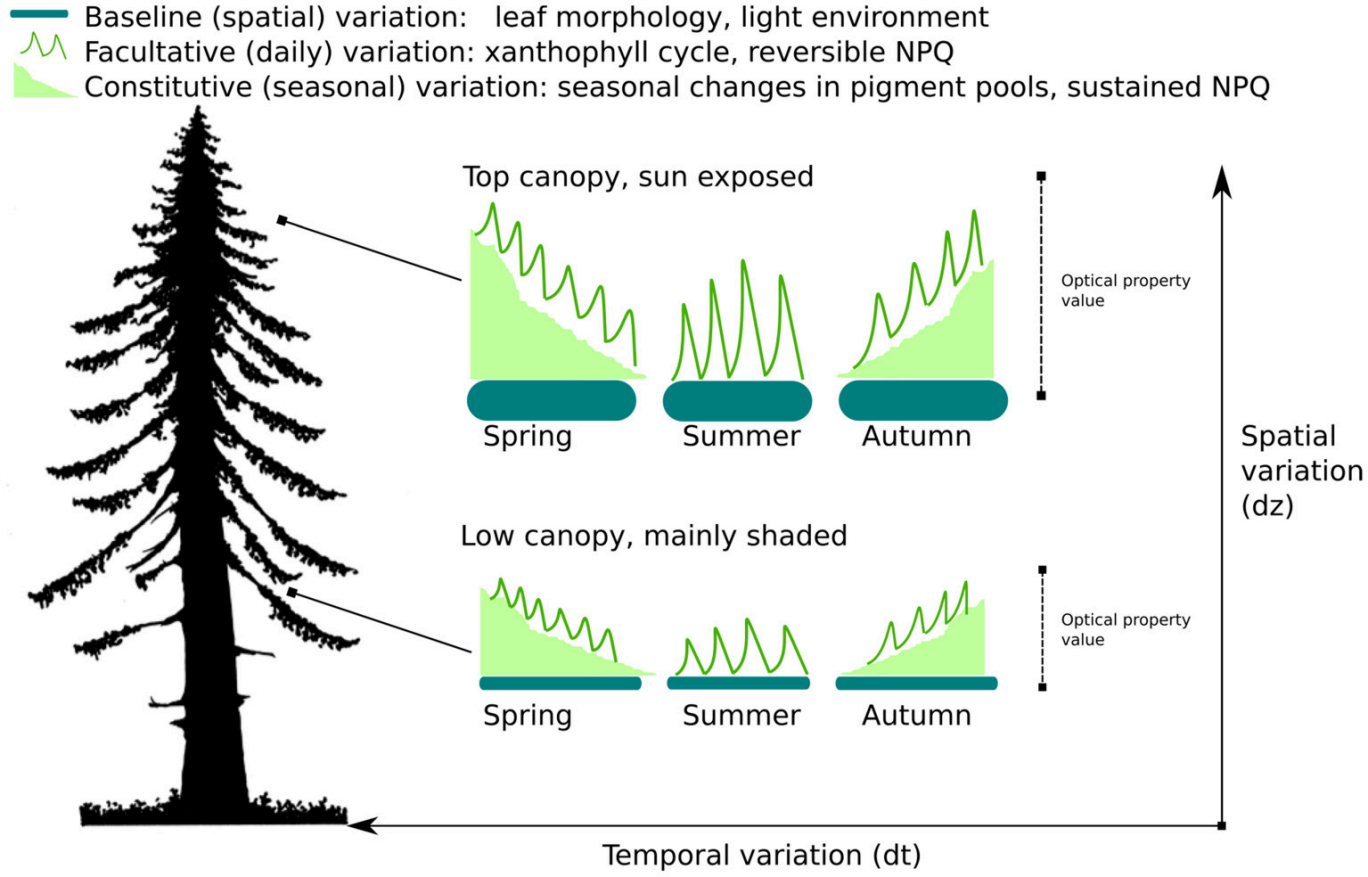
**Temporal and spatial variation of photosynthetically-related Leaf Optical Properties (LOPs)**. The diagram illustrates 3 types of variation: baseline, facultative, and constitutive. Both facultative and constitutive variation are temporal forms of variation. Baseline variation is variation in optical properties that appears constant over time, but varies across 3 dimensional space both within (as illustrated here) and across species.

We define baseline variability as the variation in LOPs and derived spectral indices (e.g. PRI) that is (mostly) constant on the seasonal timescale, but varies across 3 dimensional space both within and across species. Examples of traits that contribute to baseline variability include morphological variables such as Specific Leaf Area (SLA) and leaf thickness. If unaccounted for, baseline variability has the potential to confound and disrupt remote sensing estimates of photosynthesis or pigments. More specifically convergent relationships across space and species between LOPs and photosynthesis may be the result of the mixing of multiple baselines rather than any assumed functional relationship between productivity and optical signals. For a graphical illustration of the concept of baseline variability of LOPs see Figure 1.

The most obvious cause of baseline variability of LOPs across space is species. This variation occurs because LOPs are sensitive to not only light absorption by pigments but also to internal light scattering effects, which vary with species-dependent internal cell structure (Gausman and Allen, 1973; Govaerts et al., 1996). There are also subtler causes of baseline variability, such as gradients in (localized) light environment. Specifically, sun exposed leaves are typically more massive (Ellsworth and Reich, 1993; Lichtenthaler et al., 2007) and contain elevated levels of photoprotective pigments (Gamon and Berry, 2012) in comparison to their shaded counterparts; both of these factors affect LOP values. Additionally, leaf surface properties such as cuticular waxes have also been shown to influence LOPs (Pfündel et al., 2008; Olascoaga et al., 2014). Clearly the impact of baseline spatial variability on LOPs requires empirical investigation and quantitative characterization.

We focused our investigation on three spectral index groupings which we used to summarize spectral variation in LOPs, and which are mechanistically inter-linked via the light dependent reactions of photosynthesis. These are: 1. The PRI; 2. spectral chlorophyll fluorescence indices; and 3. the Green Normalized Difference Vegetation Index (GNDVI), which we used as a proxy of wavelength integrated photosynthetically active light absorption (Gitelson et al., 1996). The PRI was originally conceived to measure changes in the violaxanthin (Vx) cycle (Gamon et al., 1992), a regulatory mechanism that dissipates potentially harmful excess light energy as heat. In addition to thermal mechanisms such as the Vx cycle [collectively termed Non-Photochemical Quenching (NPQ)], absorbed light energy is utilized by two other competing pathways: chlorophyll fluorescence and photosynthesis. Resultantly, the combination of remotely sensed measurements of PRI, chlorophyll fluorescence, and light absorption has the potential to improve our capacity to estimate the final pathway, photosynthesis, from space. However, the combined use of these signals also raises the following question: do the same factors that control spatial variation of baseline PRI also influence chlorophyll fluorescence and photosynthetically active light absorption?

Recent studies have focused on disentangling the various temporal scales that contribute to changes in the PRI and SIF signals at the canopy scale in the case of PRI (Hmimina et al., 2015; Wong and Gamon, 2015), and at the landscape scale in the case of SIF (Yang et al., 2015). In the field, work has also begun to link spatial variation in the PRI (Gamon and Berry, 2012) and SIF (Van Wittenberghe et al., 2015) to variability in pigment content and morphology. Despite the progress resulting from these studies, the impact that species, via leaf morphology, and light environment, via morphology and pigment pool adaptation, exerts on the spatial variation of SIF and the PRI has not been explicitly separated and evaluated.

The goal of this study was to investigate the spatial variability of selected LOPs within a boreal forest. We focused our analysis on uncovering how species and light environment contributed to this variation. We measured in the boreal forest, of particular importance as the boreal forest covers almost one third of the global forested area, and due to its high-latitude location is particularly vulnerable to accelerated warming (Gauthier et al., 2015). Our main hypothesis was that factors relating to light environment and species control spatial variation of photosynthetically-related LOPs when measured at the baseline state. We measured at peak growing season under low light, so that variation in leaf photosynthetic and biochemical traits would be minimal and therefore, baseline variation highlighted.

## Materials and methods

### Experimental design, site, and plant material

Measurements were carried out during a single experimental campaign period, 24 June 2014–4 July 2014. An exception was canopy light extinction measurements (see Section Canopy Measurements) which were conducted the following summer. The campaign was conducted in stands in the local vicinity (<10 km distance) of the Station for Measuring Ecosystem-Atmosphere Relations II (SMEAR II), Finland (61.8474N, 24.2948E). In the 12 months preceding measurements the mean annual temperature was 5.19°C and sum precipitation was 566.64 mm.

**Figure 2:**
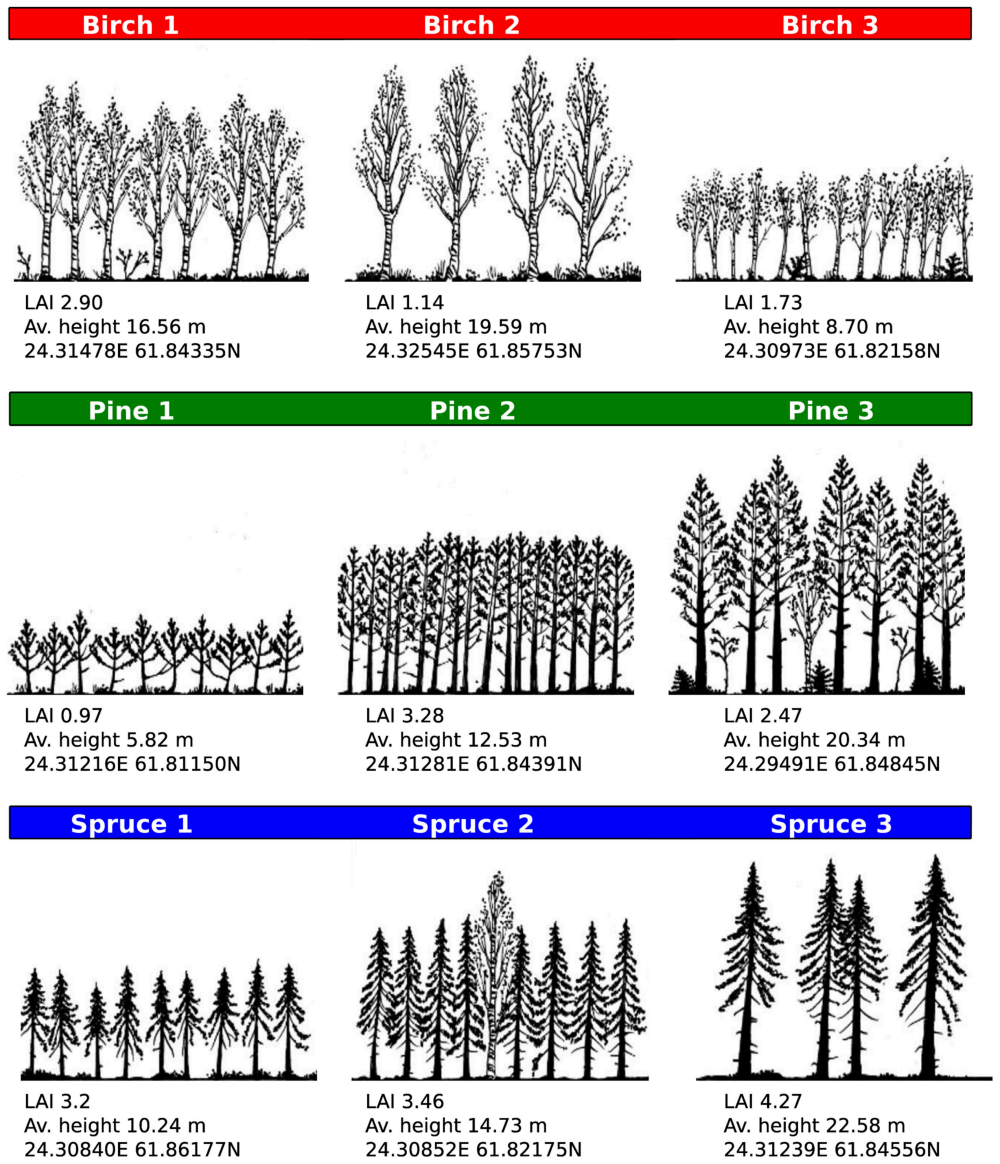
**Site characteristics and light environment**. Sites were chosen for their species and light environment diversity, as quantified by Leaf Area Index (LAI), and proximity to the SMEARII measurement station. Site characteristics were determined with a forest inventory survey conducted during the experimental period. Color coding (red = birch, green = pine, blue = spruce) is used throughout figures.

We selected a total of nine sampling sites that were dominated by tree species typical of the Finnish boreal ecosystem: Silver birch (*Betula pendula* Roth.), Scots pine (*Pinus sylvestris* L.), and Norway spruce [*Picea abies* (L.) Karst]. Each species was represented with three sampling sites (Figure 2). The sampling sites were chosen to be representative of (structurally induced) variation in light environment. Within each plot a single tree was selected randomly and used for sampling.

Three branches were cut from the top 1 m of the canopy, which was assumed to be sun exposed. Three branches were also cut from the lower canopy (second last living whorl for conifers or lowest living branch for birch). Large trees were felled early in the morning (before 9 a.m.) using a chainsaw to provide access to top canopy branches. For smaller trees we used an extendable pair of secateurs to detach sample branches. Branches were transported within a short time window (~20 min) to the site laboratory. We did not darken samples during transport, as we were aiming to avoid stomatal closure. At the laboratory the branches were re-cut under water and placed in a water filled storage container. Measurements from conifers were collected from 1 year old needles and measurements from birch leaves from those that appeared fully developed. Gas exchange and optical measurements were conducted within 6 h of the initial branch cutting. For both gas exchange and optical measurements, every measurement was repeated 3 times. Averages and standard deviations were calculated from the repeats.

### Pigment and morphological measurements

Leaf samples were collected from cut branches and frozen in liquid nitrogen within 20 min of field sampling. Samples were stored at −80°C and subsequently analyzed for Chlorophyll a, b, and carotenoid contents after Lichtenthaler, 1987). Additionally, another set of leaf samples was collected from the cut branches for Carbon (C), Nitrogen (N), and water content (WC) estimation as well as SLA estimation. Leaf fresh weight (FW) was measured using a precision scale. Fresh leaves were then color scanned (imageRUNNER ADVANCE 2225i, Canon Inc., Tokyo, Japan) at 600 dpi for subsequent estimation of total projected area using custom software (ImageAnalyzer, by Dr. Martti Perämäki). After scanning, samples were oven dried for 48 h at 70°C and dry weights (DW) obtained. Dry leaves were stored in paper bags until analysis of C and N using the Dumas dry combustion method (varioMAX analyzer, Elementar GmbH, Hanau, Germany). WC on a fresh weight (FW) basis was estimated as (FW-DW)/FW. SLA (in cm^2^/g DW) was estimated by dividing the sample total projected leaf area by its dry weight. Leaf pigment contents were estimated on a per FW basis and converted to DW and finally projected area basis using WC and SLA.

### Gas exchange and PAM fluorescence measurements

Gas exchange and fluorometer measurements were collected using a Portable IRGA equipped with a Modulated PAM fluorometer (GFS-3000, Walz GmbH, Effeltrich, Germany). Leaf samples were clipped in the measuring chamber and left a few minutes to equilibrate at 300 μmol/m^2^/s PAR. PAR levels were subsequently adjusted as follows: 300, 100, 50, 25, 300, 600, 1,200, and 0 μmol/m^2^/s. Each light level was kept for 2 min and photosynthetic parameters averaged for 10 s at the end of each light level. The intensity of the saturating pulse was at least 8,000 μmol/m^2^/s at leaf surface as measured with a separate LiCor Sensor (LI-250A, LI-COR Biosciences). The exception was the level at zero PAR which was kept for 30 min. Stationary fluorescence yield (F′) was measured every second and a saturating light pulse was supplied at the end of each light level to estimate maximal fluorescence F_M_′. A saturating light pulse after the 30 min dark acclimation period was used to obtain the reference minimal and maximal fluorescence yields, F_0_ and F_M_, and facilitate the estimation of reversible NPQ dynamics during the light response (NPQ = F_M_/F_M_′ − 1) as well as the maximum quantum yield of PSII (F_V_/F_M_). NPQ and net CO_2_ exchange (A) were derived for each light level stated above.

We used a rectangular-hyperbolic function (Kolari et al., 2014) to model A as function of PAR, for the purpose of estimating photosynthetic light response curve parameters, which we designated as photosynthetic traits:

(1) $$\begin{array}{l} A(PAR)=\frac{1}{2\theta }(\alpha PAR+A_{max}\\ \;-\;\sqrt{{\left(\alpha PAR+A_{max}\right)}^{2}-4PAR\theta \alpha A_{max}}-R_{d}) \end{array}$$

Parameters (traits) θ, *A*_*max*_, α, and *R*_*d*_ are the curvature parameter, the maximum quantum yield of photosynthesis, the light saturated rate of photosynthesis and the dark respiration rate respectively. We estimated θ, *A*_*max*_, α, and *R*_*d*_ by fitting Equation (1) to the measurements of A at the above PAR levels, using the *optimize.leastsq* method (an Levenberg-Marquardt algorithm; Marquardt, 1963) from the Scipy Python package.

### Optical measurements

Spectral measurements of LOPs were collected using a FluoWat leaf clip (Alonso et al., 2007) attached to an ASD handheld spectrometer (PANlytical Inc., Boulder, USA). The FluoWat clip utilized a short-pass 650 nm filter (650 nm OD 4, shortpass filter, Edmund Optics, U.K.). The filter was first placed in front of the illumination source light path (“filter on” position), enabling simultaneous measurement of visible reflected radiance and spectral chlorophyll fluorescence. By removing the filter (“filter off” position) after the visible reflectance/fluorescence measurements were completed it was possible to also measure near-infrared reflected radiance. For evergreen species, we constructed 1 needle thick “needle mats,” where needles were arranged laterally next to each other with small gaps, taking care not to touch the needles. The gap fraction, estimated as the proportion of needle sample to gaps between needles, was estimated using scanned images of the needle mats and a custom Python script.

We illuminated the samples with relatively low levels of PAR (<300 μmol/m2/s), using a broad-spectrum halogen bulb connected to a controllable power source (Manson EP613, Hong Kong). We measured spectra for several minutes, firstly in the filter on position and then subsequently in the filter off position. For steady state fluorescence and (visible) reflected radiance spectra we used only the final 25 spectra from the filter on period, to minimize the effects of Kautsky dynamics. Bidirectional reflectance factors were calculated by normalizing steady state leaf radiance spectra to radiance reflected from a Barium Sulphate Lambertian panel (Gigahertz-Optik GmbH, Türkenfeld, Germany) measured under identical illumination conditions. Fluorescence spectra were smoothed using a 1D Gaussian filter, and normalized by the sum of the radiance of the white panel to calculate spectral fluorescence yields.

We calculated the PRI from the (filter on) steady state reflectance spectra as:

(2) $$PRI=\;\frac{R_{531\;nm}-\;R_{570\;nm}}{R_{531\;nm}+\;R_{570\;nm}}$$

The GNDVI was calculated (using “filter off” spectra) as:

(3) $$GNDVI=\;\frac{R_{780\;nm}-\;R_{550\;nm}}{R_{780\;nm}+\;R_{550\;nm}}$$

From the normalized spectral fluorescence measurements we calculated red (F_690_) and far red emissions (F_740_) and the ratio of fluorescence in red to far red bands (F_690_/F_740_) and the sum of fluorescence across emission wavelengths (F_sum_).

By design the Fluowat instrument necessitates the use of a thin white panel to estimate incident light as reflected radiance (as described above). Because of the thinness constraint, the panel transmits a small amount of incident light. We estimated transmitted light through our panel as ~4% of the total amount of light reflected by the same panel, in the wavelength range 400–700 nm. We also found a slight wavelength variation to the transmission when compared to reflectance, which was ~1% in the 400–700 nm range. We assumed these effects had negligible impact on the comparative analysis presented here and left our spectra uncorrected.

### Canopy measurements

Leaf Area Index (LAI) was estimated from hemispherical photographs taken with a Panasonic Lumix DMC-GH1 camera (Panasonic Corporation, Kadoma, Japan) and Sigma fisheye 4.5 mm and 1:2.8 aperture lens (Sigma Corporation, Rokonkoma, NY, USA). Hemisfer software (Schleppi et al., 2007) was used to calculate LAI values from the images and pixels were divided into sky and canopy using threshold values that were checked manually.

Canopy light interception (Δ light) was estimated using an Li-190 (LICOR, Nebraska, USA) quantum sensor connected to a Li-250A light meter. First we estimated PAR under the canopy around the sampling point (PAR_*UC*_). PAR_*UC*_ was measured in a cross sampling scheme centered on the tree used for leaf traits and optical measurements, with 5 repeat measurements in direction North to South and 5 repeat measurements in the direction East to West. Then we measured (5 repeats of) PAR in an open and non-obstructed place, such as an access road, in similar sky conditions and as close to the original sampling point as possible (*PAR*_*OPEN*_). We calculated Δ light using averaged repeat measurements as:

(4) $$\Delta \;\text{ light}\;=\frac{{\text{PAR}}_{UC}}{PAR_{OPEN}}$$

### Data analyses

Our main hypothesis was that baseline spatial variability in LOPs was controlled by light environment and species. We aimed to test this by conducting linear regression analyses between LOPs and leaf morphological, biochemical, and photosynthetic traits (hereafter referred to as the LMBP traits dataset).

Prior to conducting the regression analyses, we applied a one-way ANOVA test to identify differences in every measured variable grouped by (1) canopy position (top/low) as a proxy for light environment and (2) species (Birch/Pine/Spruce). The null hypothesis (no difference between groups) was rejected if the calculated statistic was less than or equal to a family wise error rate of 0.05. If we found a significant difference in the species test, Tukey's honestly significant difference (HSD) for multiple comparisons was used to identify which species were different from each other.

We expected there to be significant co-dependence between LMBP traits, reflecting the underlying hypothetical causes of baseline variation (light environment and species). Therefore, in addition to a step-wise linear regression approach, i.e., using single variables from the LMBP data-set to model LOPs, we also conducted a Principal Components Analysis (PCA) linear regression analysis. The PCA was performed on the LMBP dataset, and then the LMBP Principal Components (PCs) were used to (linearly) model LOPs. By conducting the PCA, we aimed to assign the primary (directions of) variation in the LMBP dataset to the hypothesized background causes, principally light environment and species. Data analysis was performed in the Python programming language. PCA was run using the SKLearn (Pedregosa et al., 2011) module. The statsmodels (Seabold and Perktold, 2010) module was used for performing Tukey's HSD test and SciPy for ANOVA and linear regression.

## Results

### Leaf morphological, biochemical, and photosynthetic (LMBP) traits

**Figure 3:**
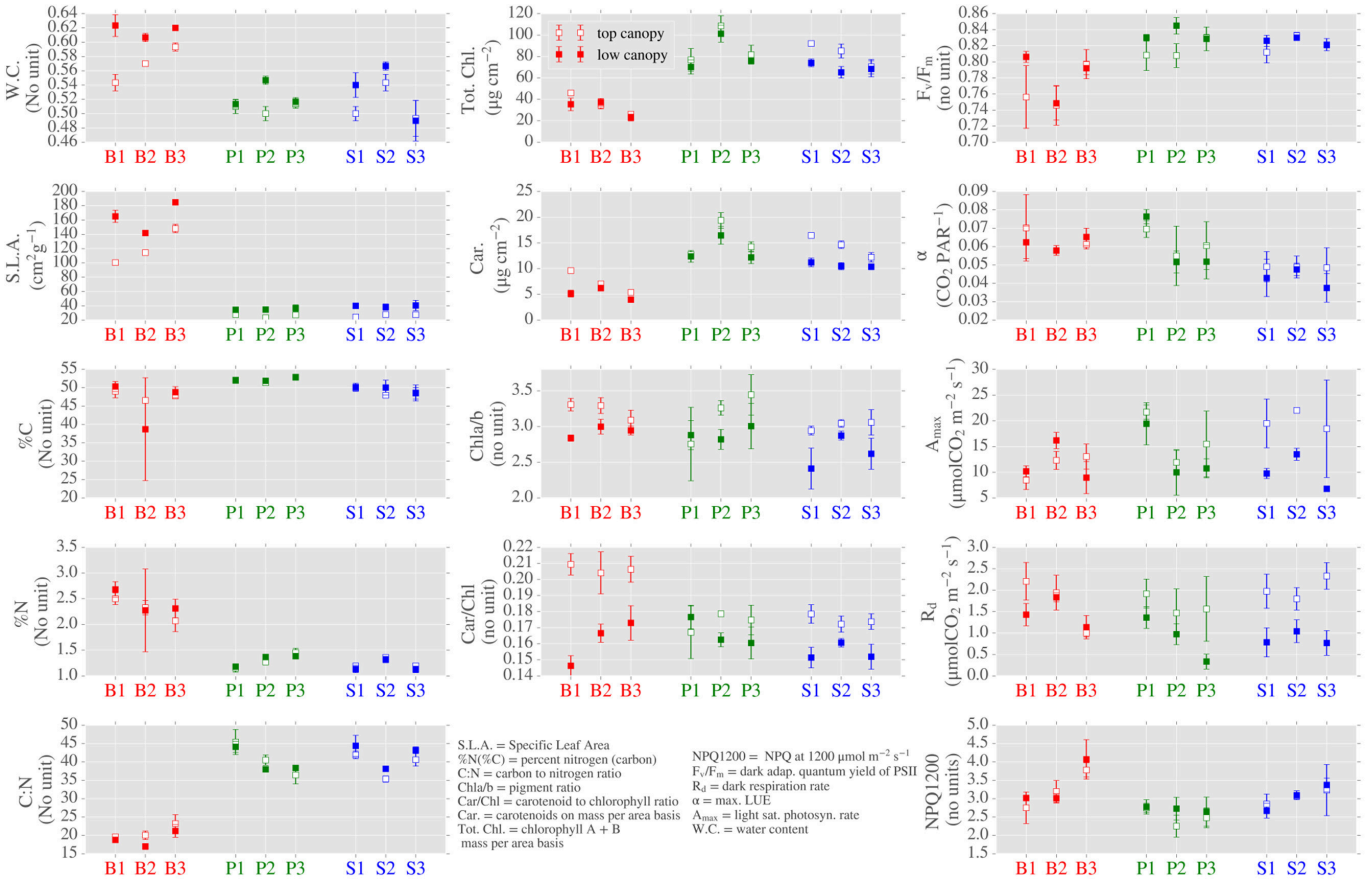
**Distributions of leaf morphological, biochemical and photosynthetic leaf (LMBP) traits across canopy position, species and sites**. Center points are means of 3 repeated measurements and the error bars show mean ± 1 standard deviation.

We found significant differences in several LMBP traits when grouped by: (1) canopy position (as a proxy for light environment) and (2) species. When grouped by canopy position, the photosynthetic traits *A*_*max*_ and *R*_*d*_ presented significant differences (Figure 3 and see Supplementary Table 1 for p values). This was also true for the ratio of chlorophyll to carotenoids (Car/Chl) and the ratio of chlorophyll *a* to chlorophyll *b* (Chla/b) which were elevated in top canopy leaves, as was carotenoid content (Car) expressed on an area basis. Water content (WC) was also found to be elevated in lower canopy leaves.

We further highlight the role of light environment on LOPs by comparing individual sites with contrasting site architecture. In particular, spruce site 2 (S2) and pine site 1 (P1) are sites with contrasting dominant species and canopy structure. S2 was dominated by spruce (97% spp. composition) with an average height of 14.73 m and canopy LAI of 3.46, whereas P1 was pine dominated (97%) with smaller trees (average height of 5.82 m) and much lower LAI of 0.97. In terms of LMBP traits, S2 presented higher Car/Chl and Chla/b ratios in top canopy leaves when compared to low canopy, this which was reversed for site P1 (Figure 3). For both sites, SLA was higher in the low canopy leaves. The photosynthetic trait *A*_*max*_ was higher in top canopy leaves at both sites, though the difference between positions was much larger for site S2.

Of the 14 traits analyzed, 12 showed significant differences when grouped by species. The majority of these differences were between the evergreen and birch sites (Figure 3 and Supplementary Table 1). Prominent examples include SLA, which was much higher in birch leaves. In terms of biochemical traits, the carbon to nitrogen ratio (C:N) was significantly lower in birch than evergreens. On a per area basis, chlorophyll content (Tot. Chl.) was also lower in birch. The photosynthetic parameter F_V_/F_M_ was elevated in the evergreens compared to birch, however, neither *A*_*max*_ nor *R*_*d*_ were distinguished by species. Differences between pine and spruce were found for α and pigment contents Car. and Tot. Chl. and also for NPQ measured at a PAR level of 1200 μmol/m^2^/s (NPQ1200).

### Leaf optical properties (LOPs) and spectral indices

**Figure 4:**
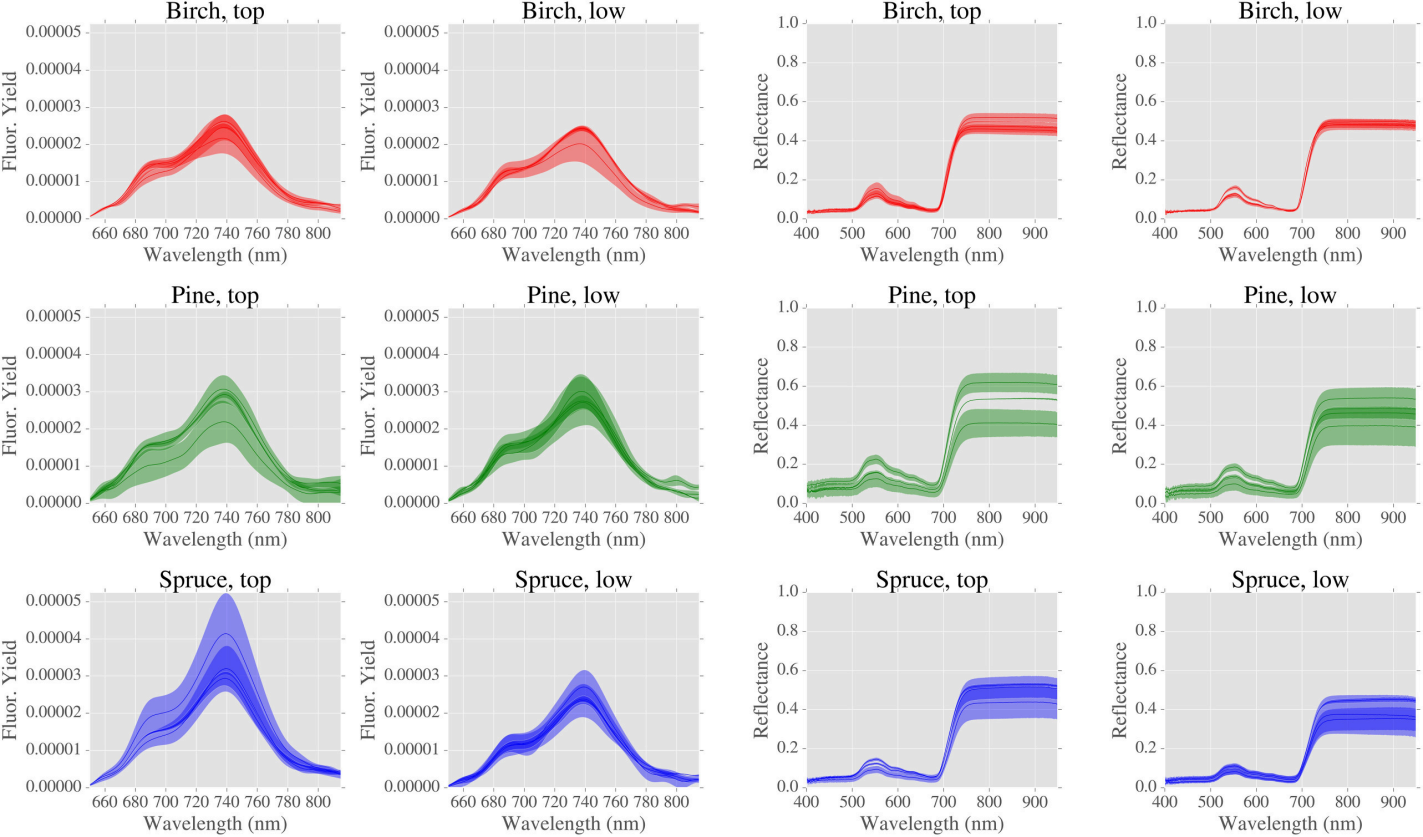
**Fluorescence and reflectance spectra (LOPs) grouped by canopy position and species**. Bold lines are means and shaded areas are mean values ± 1 standard deviation, where number of spectra is typically 3.

**Figure 5:**
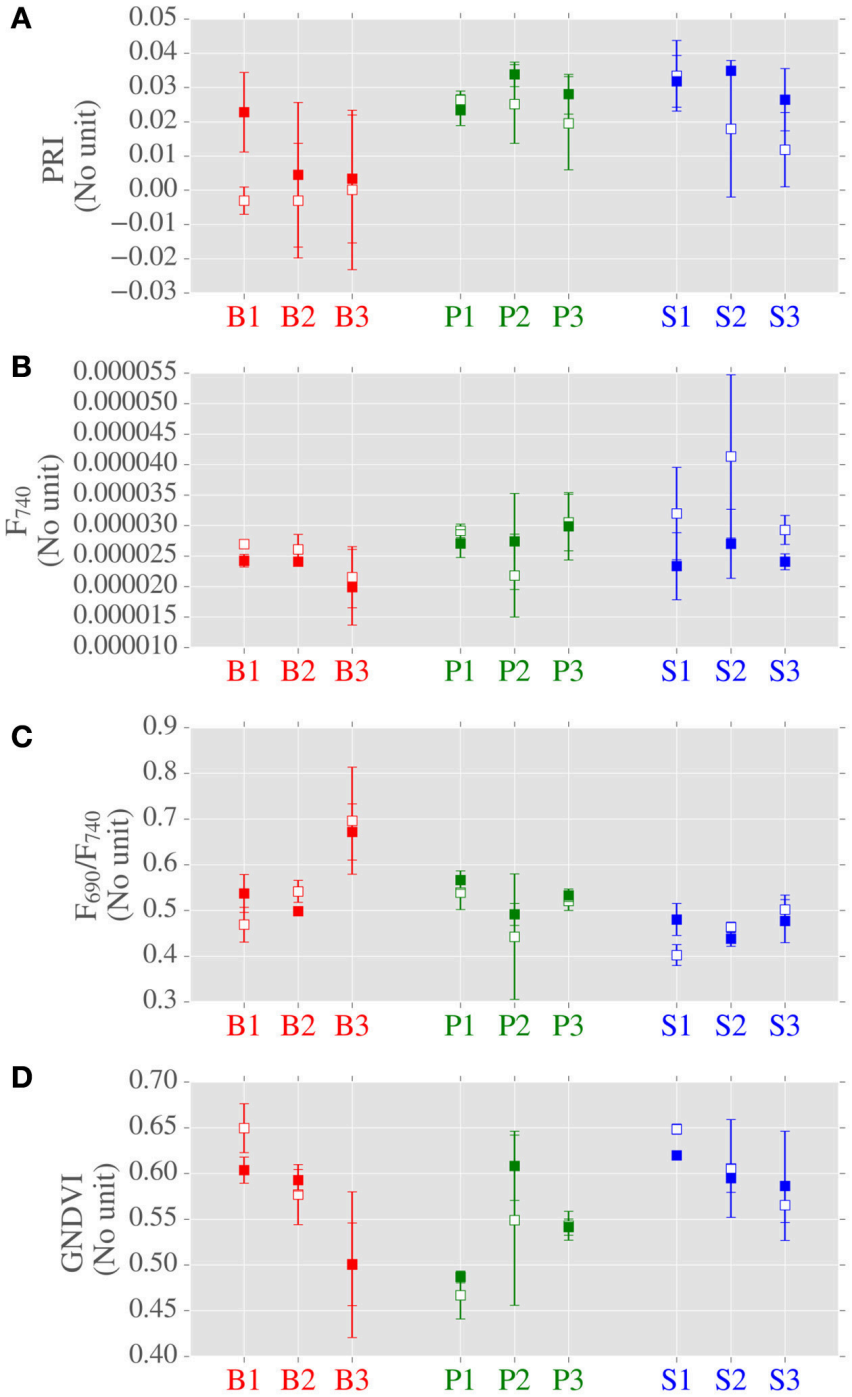
**Data distributions of photosynthetically-related spectral indices derived from LOPs across canopy position, species and site**. Subplots from top: **(A)** PRI; **(B)** fluorescence at 740 nm (F740) (normalized by incident irradiance); **(C)** ratio of red to near-infrared fluorescence (F690/F740); **(D)** GNDVI. Center points are means of repeated measurements and error bars are mean ± 1 standard deviation, where number of LOPs is typically 3.

Reflectance and fluorescence spectra are shown in Figure 4. Significant differences were found for LOPs when grouped by: 1/canopy position and 2/species (Figure 5).

When considering canopy position, fluorescence at 740 nm (F_740_) was greater for top canopy leaves than the lower canopy (Figure 5 and Supplementary Table 1). However, this was not the case at shorter wavelengths, as no significant difference due to canopy position was found for F_690_. When considering all of the sites, no significant differences were found in PRI due to canopy position. When considering LOPs at the two highlighted sites, we observed elevated PRI values in the lower canopy at the site with higher LAI (S2, LAI = 3.46) but not at the more open site (P1, LAI = 0.92). F_740_ was elevated for both sites in top canopy leaves, and as with the photosynthetic trait *A*_*max*_, the difference in canopy positions was much bigger for S2 than P1.

Next we considered the influence of species on LOPs. For the PRI and the ratio of red to far red fluorescence (F_690_/F_740_) significant differences were found between birch and evergreen species (Figure 5 and Supplementary Table 1). For GNDVI, pine was different to spruce and for F_740_ birch was different to pine but not to spruce. In addition to GNDVI, we also measured (wavelength integrated) light absorption by combining reflectance and transmittance measurements from two different instruments (FluoWat and ASD integrating sphere). We chose not to use the absorption data in the final analyses as some data were missing, however there was a clear relationship between GNDVI and absorption which increased our confidence in GNDVI as a measure of absorption (Supplementary Figure 1).

### Correlation and regression analyses

**Figure 6:**
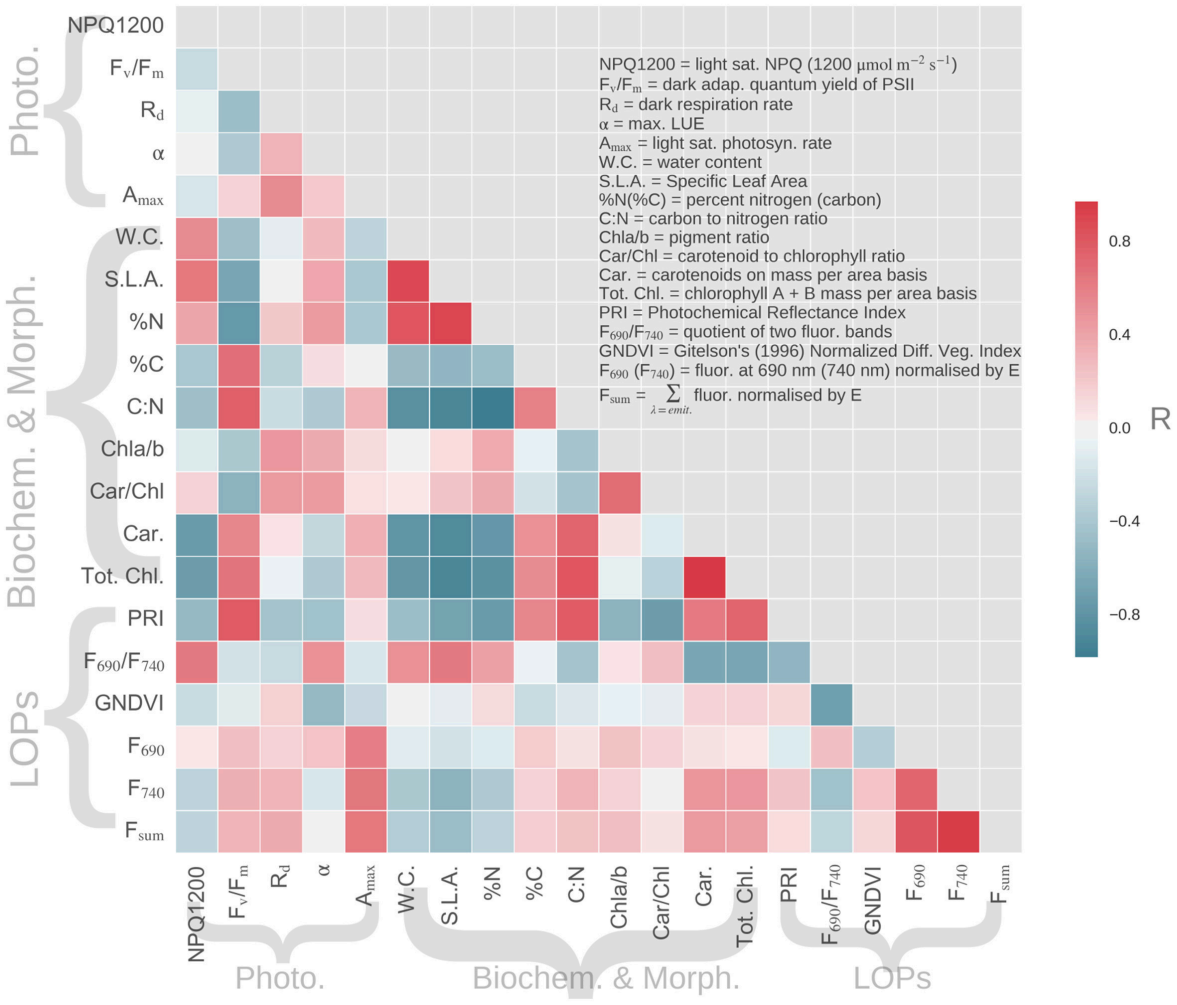
**Correlation matrix for LMBP traits and spectral indices derived from LOPs, where the number of samples per measurement is 18**. Variables are grouped by type where Photo, photosynthetic traits; Biochem. and Morph., biochemical and morphological traits; LOPs, Leaf Optical Properties.

**Figure 7:**
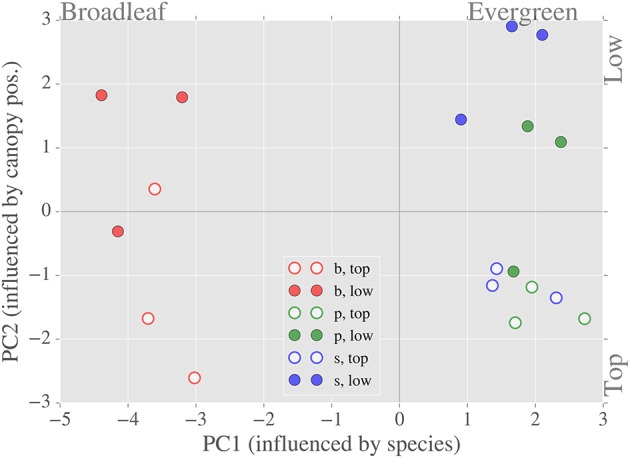
**Relationship between the first two principal components of the LMBP dataset**. Separation by species occurs along the x-axis (PC1)—variables that contribute most to PC1 include specific leaf area and total chlorophyll content. Separation by canopy position occurs along the y-axis (PC2)—variables that contribute most to PC2 include dark respiration rate and the ratio of carotenoid to chlorophyll content.

**Figure 8:**
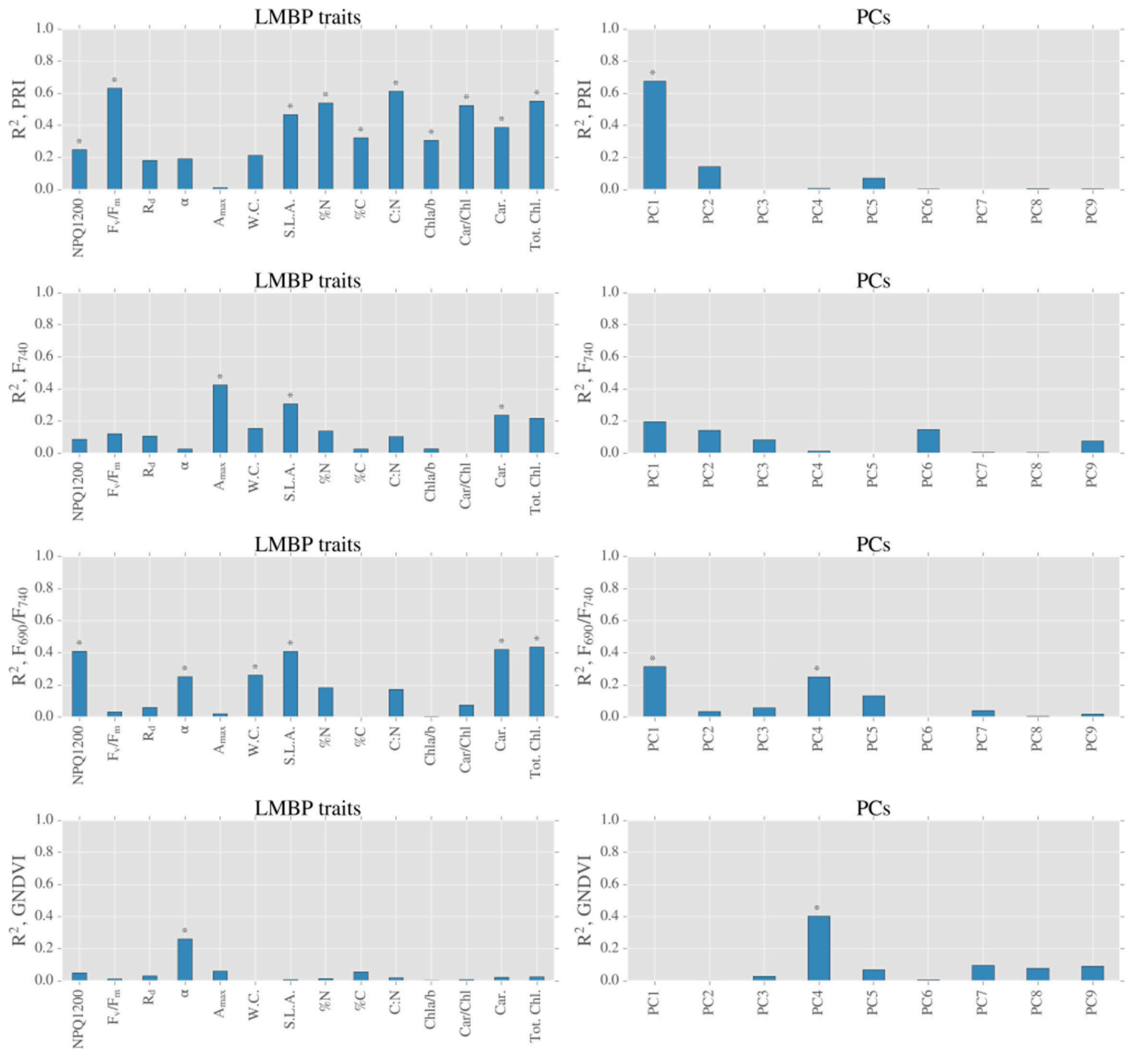
**Summary of linear models between spectral indices derived from LOPs and LMBP traits (left hand side graphs) and spectral indices derived from LOPs and PCs of LMBP dataset (right hand side graphs)**. Blue bars represent coefficients of determination for all measurements, and stars above bars (^*^) represent *p* ≤ 0.05.

In addition to calculating correlation coefficients between variables (Figure 6), we also conducted stepwise and PCA regression analyses (Figures 7, 8) to elucidate the role of the hypothetical controls of light environment and species on the measured data.

LMBP traits that were sensitive to canopy position included the pigment ratios Car/Chl and Chla/b, which also showed similar correlation trends when related to other variables (Figure 6). For example, both Car/Chl and Chla/b were moderately related to the PRI (*R* = −0.72 and *R* = −0.55, respectively). The PRI was also moderately to strongly related to the photosynthetic trait F_V_/F_M_ (*R* = 0.79). F_740_, which was identified as being sensitive to canopy position across species, was moderately correlated with the photosynthetic parameter *A*_*max*_ (*R* = 0.65) but showed close to zero correlation with the pigment ratios Car/Chl (*R* = −0.02) or Chla/b (*R* = 0.16). LMBP traits that were sensitive to species included SLA, which was related to a large number of variables including Tot. Chl (*R* = −0.91) and F_V_/F_M_ (*R* = −0.65). SLA was also correlated with the PRI (*R* = −0.68), F_690_/F_740_ (*R* = 0.64) and F_740_ (*R* = −0.0.55). Interestingly, GNDVI was correlated with very few of the LMBP traits. The exceptions were moderate correlation with α (*R* = 0.51) and moderate inter-correlation with F_690_/F_740_ (*R* = −0.71). GNDVI was also moderately correlated with gap fraction (data not shown, *R* = 0.61).

We expected significant covariation of LMBP traits due to the hypothesized causes of spatial variation, namely species and light environment. Therefore, we used a PCA of the LMBP trait dataset to summarize this covariation. Together the first and second principal components (PC1 and PC2, ordered by % variance explained) explained ~70% of the variance in the LMBP dataset. The graph of PC1 vs. PC2 shows clear separation of species on the x-axis (PC1) and separation by canopy position (PC2) on the y-axis (Figure 7). The major contributors to PC1 were WC, SLA, %N, C:N, Tot. Chl., and Car. (see Supplementary Figure 2 for PC loadings). *R*_*d*_ and the pigment ratios Car/Chl and Chla/b were the major contributors to PC2. Of the lesser PCs, *A*_*max*_ and %C contributed the bulk of PC3, whereas α as well as *A*_*max*_ were the major contributors to PC4. In summary, PC1 was representative of species-related variation in the LMBP dataset whereas PC2 reflected canopy position related variation.

We completed our analysis by analyzing the relationships between PCs and spectral indices derived from LOPs (Figure 8). The species related component, PC1, was strongly related to the PRI (*R*^2^ = 0.68, *p* < 0.05) and also moderately related to F_690_/F_740_ (*R*^2^ = 0.31, *p* ≤ 0.05). Across all sites, the light environment component PC2 was not significantly related (at the 5% level) to the PRI, or any other spectral index tested. There was very little correlation between either of the first two PCs and GNDVI, however there was moderate correlation between GNDVI and a lesser principal component PC4 (*R*^2^ = 0.40, *p* ≤ 0.05).

## Discussion

We defined baseline variation of LOPs as variation that is unrelated to temporal changes in photosynthetic and biochemical traits; therefore, baseline variation occurs across space rather than time (Figure 1). We hypothesized that there were two principal controls of spatial baseline variation for LOPs and derived spectral indices at our boreal site, species and light environment. As with LMBP traits, we found evidence that both species and canopy position influenced the spatial variation of LOPs. However, we also found that the relative importance of specific influences were dependent on the LOP or spectral index in question.

### Variability of LMBP traits

Slow growing evergreen trees typically produce structurally massive needle-like leaves, which last for multiple seasons (Givnish, 2002). In contrast, deciduous boreal species, such as the birch trees under consideration here, produce short-lived and nitrogen rich broad-leaves (Wright et al., 2004; Niinemets, 2010; Niinemets et al., 2015). Hence, morphologically distinct evergreen needles and broad-leaved birch occupy opposite ends of the leaf economics spectrum (Wright et al., 2004). The obvious difference in leaf morphology between the evergreen and birch samples was reflected by SLA values—we observed a more than three-fold increase in SLA for birch when compared to the evergreen species (Figure 3). SLA was correlated with a number of variables, including %N and TotChl, that were also sensitive the evergreen-broadleaf divide (Figure 6). Resultantly, SLA had the largest contribution by weight to PC1, which summarized trait sensitivity to species (Figure 7), and also accounted for the majority of spatially-driven variance in the LMBP dataset. This meant the morphological trait divide between evergreen and broadleaf functional type, was the main factor controlling variation in the LMBP dataset. After species, canopy position was the next most important factor. PC2, which was strongly related to the vertical change in PAR flux through the canopy (Supplementary Figure 3), summarized this variation.

Trees adapt to canopy light gradients via morphological and biochemical plasticity of leaves along such gradients (Niinemets, 2010). We found evidence of biochemical plasticity of leaves in the pigment ratios Car/Chl and Chla/b, which were sensitive to canopy position regardless of species (Figure 3). For Car/Chl, enhanced upper canopy values were due to the accumulation of photo-protective carotenoids, an adaption to high light. (Lichtenthaler et al., 2007). Enhanced upper canopy Chla/b likely reflected a reduction in the number of light harvesting complexes (containing chlorophyll b), also an adaptation to high light (Anderson et al., 1995; Kitajima and Hogan, 2003). In addition to biochemical plasticity, we found evidence of photosynthetic adaptation to canopy position. Higher dark respiration rates (*R*_*d*_) were observed in the upper canopy, consistent with increased turnover rates found in light adapted leaves (Lusk and Reich, 2000). In summary, the main contributors to the light environment related principal component, PC2, were the pigment ratios Car/Chl and Chla/b and the rate of dark respiration, *R*_*d*_.

We attempted to separate out the spatial variation of traits related to species from that related to light environment. However, both trait and LOP variation across space is likely due to a combination of interacting causes. Correspondingly, several traits were sensitive to both canopy position and species. For example, SLA was sensitive to species but also presented elevated values in the lower canopy; the same was true for water content. These differences are evidence of adaptations to gradients in light environment, as increased light exposure correlates with thicker, moister leaves (Lichtenthaler et al., 2007; Niinemets, 2010). Finally, although we measured raised *R*_*d*_ levels in the upper canopy for all species, this result was not the case for other photosynthetic traits such as *A*_*max*_ and F_V_/F_M_. This was because both *A*_*max*_ and F_V_/F_M_ presented lower values for birch leaves, regardless of position, when compared to evergreens. This was unexpected, as birch leaves usually have higher photosynthetic capacity in comparison to evergreens (Pumpanen et al., 2009). This seemingly contradictory result could be explained by the prevailing weather at the time of the campaign, which was unseasonably cold, potentially preventing the full development of birch leaves at the sampling time. In summary, analysis of the LMBP dataset revealed strong underlying control by species and light environment. We expected these controls to be the primary causes of baseline variation of LOPs across space.

### Baseline variability of LOPs and spectral indices

The transfer of radiation through leaves is determined by not only the absorption of light by photosynthetic pigments but also by internal scattering, which occurs at cell walls and is therefore sensitive to species specific leaf morphology (Gausman and Allen, 1973; Govaerts et al., 1996). Therefore, the strong correlation that was found between the PRI and the species related PC1 (Figure 8) likely reflects the fact that baseline spatial variability of the PRI is determined by species specific internal morphology. Although, eclipsed by species related variation, PRI was also found to be sensitive to changes in canopy position (Figure 5), which was evidence of the response of PRI to gradients in light environment. This effect was emphasized by the across-species relationship found between PRI and the light environment sensitive trait Car/Chl., as previously observed (Sims and Gamon, 2002; Filella et al., 2009). We also found significant differences in PRI between upper and lower canopy foliage at sites with relatively high LAI values, a result consistent with Gamon and Berry (2012). Taken together, these results point to the significant, but not dominant, role that local light environment plays in controlling the spatial variation of baseline PRI. The main control of the spatial variation of baseline PRI was the evergreen-broadleaf divide.

The ratio of red to far red fluorescence (F_690_/F_740_) is primarily known as an indicator of light absorption (Gitelson et al., 1998; Buschmann, 2007). This is because the chlorophyll absorption spectrum partially overlaps with the fluorescence emission spectrum at shorter red wavelengths, but not at longer wavelengths. Like PRI, we found that baseline variation of F_690_/F_740_ was sensitive to the evergreen-broadleaf divide, and correspondingly we observed a moderate correlation between TotChl. and F_690_/F_740_ (Figure 8). However, in addition to chlorophyll absorption, F_690_/F_740_ could also be sensitive to changes in NPQ. Such a relationship could be due to either wavelength dependent light penetration in the leaf (Agati et al., 1995) or the wavelength dependent contribution of photosystem I fluorescence to NPQ (Porcar-Castell et al., 2014). Accordingly we found a moderate correlation between light saturated NPQ (NPQ1200) and F_690_/F_740_. However, we did not find any difference in NPQ1200 between evergreens and birch leaves, which we found for TotChl. Consequently across species differences in F_690_/F_740_ were most likely caused by differing pigment allocation strategies between broadleaves and evergreens.

Unlike F_690_/F_740_, individually neither F_740_ nor F_690_ were separable by the evergreen-birch functional divide. Also in contrast to F_690_/F_740_, the baseline variation of F_740_ was sensitive to canopy position. More specifically, F_740_ was elevated in the upper canopy for each species tested. Light environment induced gradients in pigment pools could be partially responsible for elevated far red fluorescence. This is because an increase in chlorophyll, as was observed in upper canopy leaves, is typically accompanied by a coincident increase in fluorescence emission. However, light re-absorption at shorter red wavelengths has the potential to counteract any potential gains due to increased emission (Buschmann, 2007; Pedrós et al., 2010). The fact that re-absorption is many times weaker at longer wavelengths could explain why we observed a significant effect of canopy at longer wavelengths only. Elevated top canopy F_740_ could also reflect the influence of light environment on photosynthetic functionality, as a significant and positive relationship was found between F_740_ and A_*max*_, a trait influenced by canopy position. This result strengthens the case for the use of far red fluorescence as a general (across species) indicator of photosynthetic performance. Finally, although we found no significant effect of gap fraction on fluorescence spectra in this study, we expect at least some inter-needle reabsorption to occur in the red region. We therefore, recommend that care is taken to use a standardized protocol when measuring needle-leaf fluorescence spectra, and that gap fractions are also recorded (as in this study).

GNDVI was formulated by Gitelson et al. (1996) as a measure of light absorption that could be scaled from the leaf to the satellite. We found a strong relationship between light absorption and GNDVI (Supplementary Figure 1), but no significant influence of neither species nor canopy position. We also did not find an across species relationship between pigment contents (expressed on an area basis) and GNDVI. As a consequence, it is possible that there was a decoupling of the relationship between light absorption and chlorophyll for high chlorophyll content evergreen needles. Such an effect has important implications for remote sensing studies, as canopy scale retrievals of biochemistry rely on theoretical relationships between leaf scale light absorption and chlorophyll content (Zhang et al., 2008; Croft et al., 2013). Gap fraction effects could be partially responsible for the low correlations found between GNDVI and other traits (Figure 8), as we found a moderate effect of gap fraction on GNDVI. Although, correction of reflectance spectra using estimated gap fractions is possible, such correction is a potential source of error hence we left our spectra uncorrected in this instance (Olascoaga et al., 2016). For the LOPs under investigation in this study, the disentanglement of the effects of absorption and biochemistry from structure and morphology is a complex task that warrants further investigation using a physically-based modeling approach, such as that developed by Govaerts et al. (1996).

### Conclusions and implications for upscaling

The evidence presented in this study has important consequences for remote sensing practice. Firstly, we found that baseline variability in the PRI was mostly determined by plant species, which masked light environment related control. Further to this, the baseline variability between species was of the same order of magnitude as the variation that occurs in response to the reversible Vx cycle (Gamon et al., 1992; Atherton et al., 2016). For that reason, when considering the challenge of upscaling from the leaf to the satellite pixel, we suggest that caution should be used when interpreting remote sensing images of PRI, especially if mixed forest types are represented within a single, coarse grained pixel.

In contrast to the PRI, our results indicate that chlorophyll fluorescence is generally more conservative in terms of species related baseline variation than the PRI. The exception to this was F_690_/F_740_ which was sensitive to the evergreen-birch divide. We also found evidence of the effect of canopy light gradients on fluorescence emission at longer far red wavelengths. The relative robustness of chlorophyll fluorescence is promising for remote sensing applications, where fluorescence is measured across species and space (Joiner et al., 2013; Frankenberg et al., 2014; Guanter et al., 2014; Zhang et al., 2016).

Although we standardized on leaf scale optical properties, understanding shoot scale radiative transfer processes is equally critical to the proper interpretation of remote sensing measurements of evergreen trees (Stenberg et al., 1998). That being so, a future direction of study would highlight baseline variability of shoot level optical properties, such as single-scattering shoot albedo (Mõttus and Rautiainen, 2013) and shoot scale fluorescence emission. Finally, although we focused on spatial variation, understanding temporal variation is equally important if optical measurements are to be linked to functional traits. Recent studies (Hmimina et al., 2015; Wong and Gamon, 2015) have focused on disentangling the various processes and scales that cause temporal variation of the PRI signal. In contrast, the mechanisms controlling the seasonal variation of spectral fluorescence are less well-known (Porcar-Castell et al., 2014). In light of this, we have recently conducted a study where we measured seasonal variability of spectral fluorescence and other LOPs during the spring photosynthesis recovery period that typifies boreal type forests and our results are forthcoming (Zhang et al., in preparation).

## Author contributions

JA conducted the bulk of the optical measurements, conducted the data analyses and wrote the text. BO summarized the survey data, drew the tree illustrations used in Figures 1, 2, conducted integrating sphere optical measurements and provided comments during text revision. LA developed the FluoWat instrument that was used for the bulk of the optical measurements and provided guidance on its use for the measurement campaign. LA provided comments during text revision. AP conceptualized the study and conducted the majority of the LMBP trait measurements. AP provided substantial guidance on the writing of the text and data analyses.

## Funding

This study was funded by grants from the University of Helsinki (#490116) and the Academy of Finland (#288039, #293443, #272041).

### Conflict of interest statement

The authors declare that the research was conducted in the absence of any commercial or financial relationships that could be construed as a potential conflict of interest.
