# Supplementary material for: Spatial Variation of Leaf Optical Properties in a Boreal Forest Is Influenced by Species and Light Environment
Source: Front Plant Sci. 2017 Mar 14;8:309. doi: 10.3389/fpls.2017.00309 (PMC5349083; doi:10.3389/fpls.2017.00309)
Supplement: Supplementary file 1 [file DataSheet1.DOCX]

Supplementary Material

Spatial variation of leaf optical properties in a boreal forest is controlled by species and light environment

Jon Atherton^1^*, Beñat Olascoaga^1^, Luis Alonso^2^, Albert Porcar-Castell^1^

*** Correspondence**: jon.atherton@helsinki.fi

## Supplementary Figures


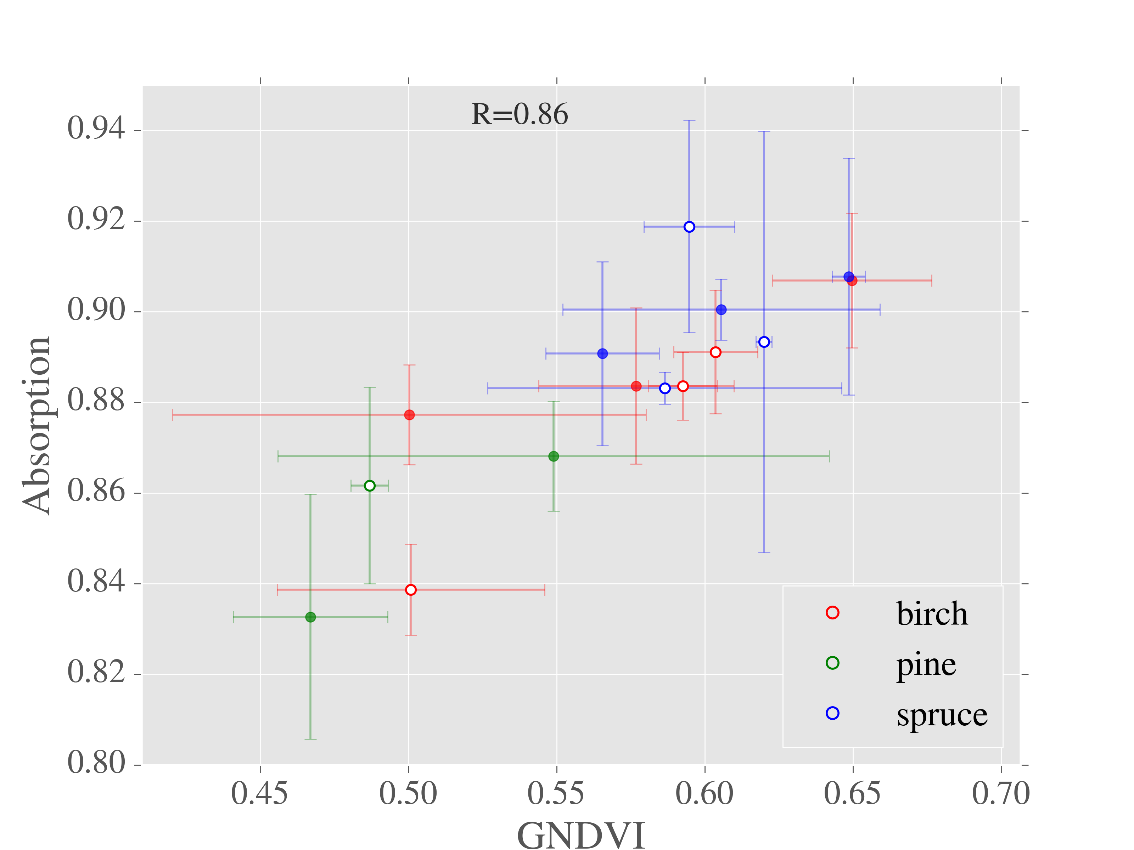


**Supplementary Figure 1.** Relationship between GNDVI and visible light absorption. Light absorption was calculated as the average value of 1 – R – T across the wavelength range 400 to 700 nm. We used GNDVI as a proxy for light absorption because our actual measurements of light absorption (shown above) were incomplete (missing values for some sites and positions). The absorption data were also potentially unreliable as we used two different methodologies to measure R and T (integrating sphere for some data and FluoWat for others).
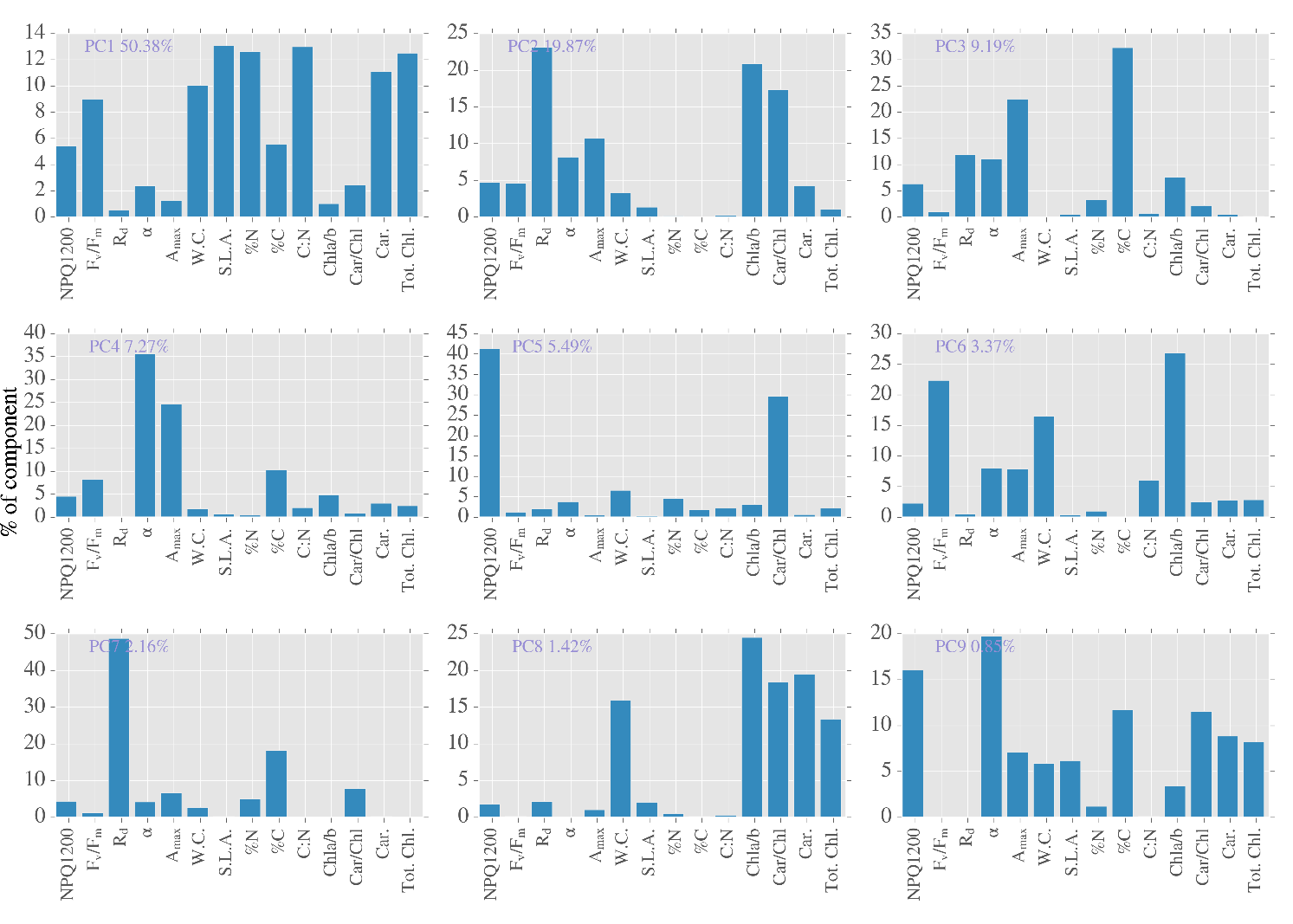
**Supplementary Figure 2.** Principal component weightings for LMBP dataset with percentage of variance explained in top left corner.


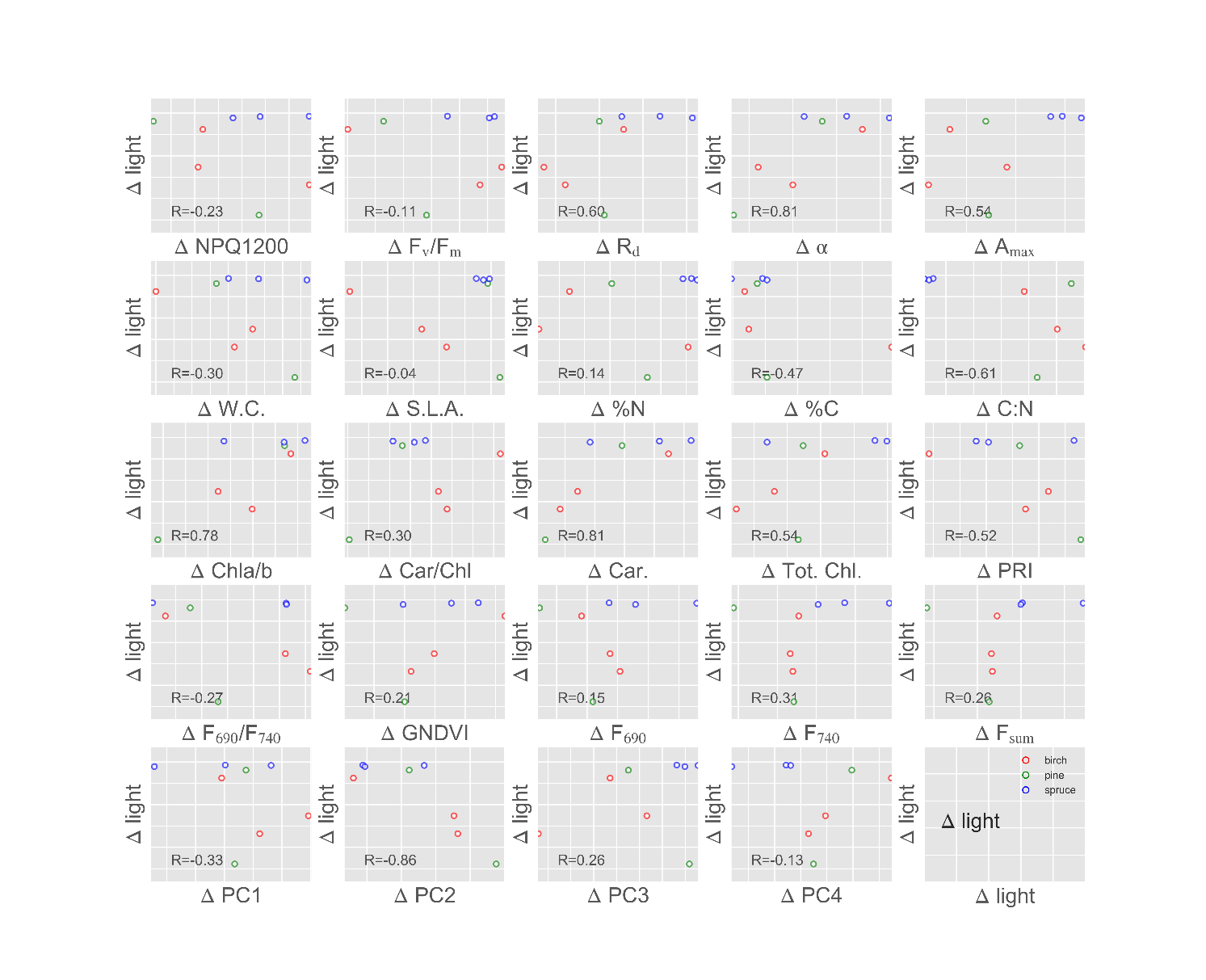


**Supplementary Figure 3.** Change in canopy light interception (Δ light) versus change in variable. Change is calculated across canopy position at each site (Δ variable = variable TOP – variable LOW) for a particular variable.

## 1.2 Supplementary tables

**Supplementary table 1**. ANOVA (Top Vs Low, B Vs P Vs S) and Tukey HSD (others) statistical test results.

|  | TOP VS LOW | B Vs P Vs S | B Vs P | B Vs S | P Vs S |
| --- | --- | --- | --- | --- | --- |
| NPQ1200 | p = 0.48 | p ≤ 0.001 | p ≤ 0.001 | p = 0.14 | p ≤ 0.05 |
| Fv/Fm | p = 0.17 | p ≤ 0.001 | p ≤ 0.001 | p ≤ 0.001 | p = 0.90 |
| Rd | p ≤ 0.001 | p = 0.27 |  |  |  |
| α | p = 0.75 | p ≤ 0.001 | p = 0.90 | p ≤ 0.05 | p ≤ 0.05 |
| Amax | p ≤ 0.05 | p = 0.13 |  |  |  |
| WC | p ≤ 0.05 | p ≤ 0.001 | p ≤ 0.001 | p ≤ 0.001 | p = 0.65 |
| SLA | p = 0.15 | p ≤ 0.001 | p ≤ 0.001 | p ≤ 0.001 | p = 0.90 |
| %N | p = 0.82 | p ≤ 0.001 | p ≤ 0.001 | p ≤ 0.001 | p = 0.63 |
| %C | p = 0.84 | p ≤ 0.001 | p ≤ 0.001 | p = 0.13 | p = 0.07 |
| C:N | p = 0.95 | p ≤ 0.001 | p ≤ 0.001 | p ≤ 0.001 | p = 0.90 |
| Chla/b | p ≤ 0.001 | p ≤ 0.05 | p = 0.85 | p ≤ 0.05 | p = 0.11 |
| Car/Ch | p ≤ 0.001 | p ≤ 0.05 | p = 0.06 | p ≤ 0.05 | p = 0.57 |
| Car. | p ≤ 0.05 | p ≤ 0.001 | p ≤ 0.001 | p ≤ 0.001 | p ≤ 0.05 |
| Tot. Chl. | p = 0.23 | p ≤ 0.001 | p ≤ 0.001 | p ≤ 0.001 | p ≤ 0.05 |
| PRI | p = 0.06 | p ≤ 0.001 | p ≤ 0.001 | p ≤ 0.001 | p = 0.90 |
| GNDVI | p = 0.64 | p ≤ 0.001 | p = 0.18 | p = 0.09 | p ≤ 0.001 |
| F690 | p = 0.09 | p = 0.52 |  |  |  |
| F740 | p ≤ 0.05 | p ≤ 0.05 | p = 0.16 | p ≤ 0.05 | p = 0.65 |
| Fsum | p ≤ 0.05 | p = 0.15 |  |  |  |
